# Perspectives of Young People on Social Media-Based Sexuality Education Using a Feminist Approach in China: A Qualitative Study

**DOI:** 10.1007/s10508-024-03015-z

**Published:** 2024-11-18

**Authors:** Yexuan Ma, Sikky Shiqi Chen, Holroyd Eleanor, William Chi Wai Wong

**Affiliations:** 1 Department of Family Medicine and Primary Care, Li Ka Shing Faculty of Medicine, The University of Hong Kong, 3/F, Ap Lei Chau Clinic, 161 Main Street, Ap Lei Chau, Hong Kong SAR, China; 2 School of Clinical Sciences, Auckland University of Technology, Auckland, New Zealand; 3 Department of Family Medicine and Primary Care, University of Hong Kong-Shenzhen Hospital, Shenzhen, People’s Republic of China

**Keywords:** Sexuality education, Gender, Feminism, Social media, China

## Abstract

The feminist approach to sexuality education challenges and changes power dynamics and inequalities embedded within sexuality, while social media serves as a tool for building online communities and raising awareness about feminist issues. Adopting a feminist approach to sexuality education, particularly through social media platforms, remains largely unexplored and underdeveloped. In China, where traditional sex education is often limited and stigmatized, sexuality has been influenced by a complex interplay of traditional gender norms, evolving social attitudes, and state intervention. The aim of this study was to evaluate both sex educators and their online followers’ perceptions of the implementation of social media-based sexuality education using a feminist approach in China. Semi-structured online interviews were carried out with 10 educators and 18 young followers, followed by thematic analysis using NVivo. The findings indicated that social media-based sexuality education using a feminist approach might be used to help disrupt oppressive norms about gender and sexuality and foster a positive attitude toward women’s sexuality. Chinese online sex educators strategically avoided the term “feminism” and creatively evaded censorship and spread their messages in alignment with government policies while advocating for gender equality. However, participants also highlighted the existence of barriers, including internal conflicts, gender imbalances, social stigmatization, and political constraints, which needed to be addressed in order to effectively implement this approach.

**Supplementary Information:**

The online version contains supplementary material available at 10.1007/s10508-024-03015-z.

## Introduction

Sexuality education has shifted from a narrow biomedical focus to a more comprehensive, holistic, and rights-based approach that recognizes sexuality as a fundamental part of human development and well-being (Chavula et al., 2022; Goldfarb & Lieberman, 2021). The biomedical-oriented programs rarely discuss sexuality in its broader sense and have contributed to a discourse that equates sexuality with danger rather than pleasure, with a focus on the perils of sexual expression for women in particular (Allen & Elliott, 2008; Jolly et al., 2013; Mark et al., 2021).

Traditional sexual scripts which portray men in the role of initiators and women in the role of gatekeepers continue to dominate how sexuality is depicted (Benoit & Ronis, 2022). These discourses have contributed to power imbalances in sexual relationships, with women often expected to prioritize men’s desires and needs, making it challenging for them to reject unwanted advances (Frith & Kitzinger, 2001). Furthermore, the construction of women’s and men’s sexuality presents a dichotomy, where women are seen as lacking erotic desire and men as disconnected from their emotional connections (Allen, 2003).

The feminist approach to sexuality education can be seen as a new way to improve physical, emotional, mental and social well-being. It points out the historical silence on women’s sexual desire and the construction of young women as passive sexual subjects (Fine, 1988; Welles, 2005), which draws our attention to unequal gendered power relationships that enable gender-based violence to be reproduced and prevent young women from making autonomous sexual decisions and developing an empowering sense of sexual self (Connell & Elliott, 2009; Edward, 2016; Harrison & Ollis, 2015). It also challenges the exclusive focus on heterosexuality in common sex educational practices and advocates for sexual diversity and deconstruction of the man/woman dichotomy (Grant & Nash, 2019; Koepsel, 2016; Measor et al., 2000; Ullman & Ferfolja, 2015).

Social media presents a new mechanism for sexuality education and a contemporary space for young adults across the world to share their concerns, knowledge, and experience, and organize collective actions on sexual rights and justice (Attwood et al., 2015; Chang & Tian, 2021; Chung, 2016; Martin & Valenti, 2013; Tsaliki, 2015; Tung et al., 2015; Yeo & Chu, 2017). Previous research has shown that feminists can take advantage of social media which provides an opportunity for creating online feminist communities, disrupting hegemonic discourses on sexuality, and developing their own discourses of sexual desire (Baer, 2016; Chu, 2017; Crossley, 2015; Harris, 2004; Muise, 2011; Wood, 2008). Social media can also be a useful tool to reduce sexual shame and regain control over sexual knowledge (Wood, 2008). By discussing various kinds of sexual identities and expression, social media approaches may also have unique potentialities to enable fluid gender enactments and a breakdown of gender binaries (Dobson & Ringrose, 2016; Johnson, 2017; O’Brien, 1999). However, of note is that the digital realm may not be completely “free” and “empowering” spaces without encumbering by pre-established sex and gender norms, and political and economic influences (Dobson & Ringrose, 2016). Feminists have been reported to be exposed to online harassment tactics and hate speech (Megarry, 2020), and subject to strict online censorship and restrictions from digital gatekeepers such as Google, Facebook, and Twitter (Johnson, 2017; Oosterhoff et al., 2017; Pearce & Rodgers, 2020).

Given significant changes in both young people’s sexual behaviors and gender politics in contemporary China, the current study drew from a diverse sample of young participants of various gender and sexual identities to explore the perceptions regarding social media-based sexuality education using a feminist approach. The research question was: What are the perceptions of sex educators and online followers regarding the use of a feminist approach in social media-based sexuality education?

### A Feminist Approach to Sexuality Education

From a feminist perspective, sexuality is not solely an individual attribute or quality that defines personhood, but rather a complex social construct encompassing various dimensions. Feminist theories differ in their conceptualization of the relationship between sex and gender, as well as the role of sex in female oppression or liberation. Since the 1970s, the question of whether and how (hetero) sexuality can be understood as an institution or practice, a source of repression, danger, and victimization, or as a domain for exploration, pleasure, and agency has been the subject of ongoing debate (Holmes, 2000; Vance, 1984). Radical feminists posited that sexuality served as a locus of man’s power, wherein women’s sexuality was owned and controlled by men, serving their interests and shaped by their perspectives (Jeffreys, 1990; Koedt, 1994; MacKinnon, 1989; Rich, 2003). In contrast, sex-positive feminists sought to reclaim and celebrate women’s sexuality, challenging the notion that women’s sexuality was inherently oppressive or degrading (Ehrenreich et al., 1987; Rubin, 1975; Willis, 1981). Liberal feminists advocated for challenging gender stereotypes and believed in equality between men and women while recognizing their inherent differences (Hall, 2015). The influence of poststructuralism and postmodernism during the 1980s and 1990s further complicated the understanding of heterosexuality, challenging its naturalization. Third-wave feminists introduced the concept of “intersectionality,” recognizing that experiences of sexuality were shaped by intersecting identities such as race, class, and ability (Crenshaw, 1995). These feminists highlight how systems of oppression and privilege impact individuals’ sexual experiences, advocating for inclusive and equitable approaches to sexuality.

### Sexuality and Sexuality Education in Post-Socialist China

After the reforms and opening up of China in 1979, China experienced a slow but steady sexual revolution in which personal pleasure was emphasized, and sexual attitudes and behaviors related to individual freedom changed (Higgins & Sun, 2007). Studies found that premarital and extramarital sex were increasingly accepted in China, particularly among young people (Parish et al., 2007; Xiao et al., 2011). The shifting attitudes toward sexuality aligned with the liberation of the market, the proliferation of individual consumerism, and the convergence of state intervention (Liao, 2020; Zhang, 2012). As a result of the economic reforms in 1979, China’s beauty industry experienced meteoric growth, propelled by mass media and consumer culture (Jung, 2018). Advertisements for beauty products encouraged women to express their sexuality and embrace conventional femininity through the use of dresses and cosmetics (Liao, 2020). Sexual desires, particularly those associated with women’s bodies, were highly commodified, increasingly linked to pleasure, and conformed to stereotypical gender expectations of Chinese society (Chen, 2016; Isherwood, 2004). The convergence of market economics and reform discourse facilitated a rapid sexualization of public discourse, characterized by the essentialization and naturalization of gender differences, such as the yin-yang balance between men and women in Chinese culture (Barlow, 2004). Simultaneously, state control over women’s bodies was reinforced through various national policies, including the “third-child policy” and a mandatory “cool-off” period for divorce, which reflected male authority figures’ calls for married women to “return home” and take on their traditional roles as reproducers and nurturers (Song, 2011).

Sexuality education in China was historically governed by the state and was highly medicalized through the dissemination of “scientific sexual knowledge” by schools (Aresu, 2009). It focused on sexual morality and ethics that aimed to transform young people into self-controlled, self-disciplined, and moral subjects (Huang, 2018). Confucianism was seen as repressing sexual desire, with any sexual expression that might threaten the stability of the family regarded as a threat to morality and social order (Sigley, 2006).

### From State-Led to Non-Governmental Engagement to Feminist Media Activism

The trajectory of feminism in China evolved in response to historical and political contexts. Confucian ethics, deeply entrenched in Chinese society, perpetuated a masculine and patriarchal framework, creating a challenging environment for women (Han, 2018). Within this framework, the dominant form of masculinity, known as hegemonic masculinity, was culturally valued and reinforced (Connell, 1993).

During the socialist era under Mao’s leadership, the All-China Women’s Federation (ACWF) served as a platform for socialist feminists to promote women's legal rights and economic benefits (Zheng, 2016). However, this state-sanctioned organization also reproduced and reinforced hegemonic masculinity within its framework. In the 1980s, the emergence of non-governmental organizations (NGOs) led by professors, lawyers, journalists who are women, and ACWF cadres gained momentum, particularly after the 1995 World Women’s Conference in Beijing (Wu & Dong, 2019). These NGOs tackled issues such as domestic violence, reproductive health rights, and rural women’s empowerment, often receiving support from official institutions and foreign agencies (Wu & Dong, 2019).

The rise of individual feminism, influenced by neoliberalism and liberal feminist ideologies, introduced notions of freedom of choice and consumerism (Rottenberg, 2014). The growing feminist movements and activism, driven by advances in digital technology, have been unprecedented in both the scope of participation and visibility, resulting in a surge of research and commentary on the new generation of feminists (Peng, 2020; Wu & Dong, 2019).

## Method

In-depth interview methods were used to explore the research question regarding the perceptions of sex educators and online followers of social media-based sexuality education using a feminist approach.

### Participants

A total of 28 participants (10 sex educators and 18 online followers) took part in a semi-structured interview related to their perceptions of social media-based sexuality education programs using a feminist approach. The reason for recruiting both sex educators and young people was that the public agenda of sexuality education should be defined by policy- makers, service providers, as well as the needs and perceptions of young people (Measor et al., 2000).

The study participants were recruited through Chinese sexuality education organizations, such as “Safeclass,” “Toughyouth,” “Vagina Project,” and “Girl Up China.” Eligible sex educators were recruited based on being the founders or co-founders of social media-based sexuality education organizations in China. The criteria for online followers included Chinese young adults aged 18–29 years old and had the experience of receiving sexuality education on social media. Individuals who were unable to read and understand Chinese were excluded.

### Procedure

Purposive sampling was adopted in recruiting both the key workers of Chinese sexuality education organizations and their online followers who had fulfilled the inclusion criteria. This sampling strategy permitted the researchers to focus on people or events that are good grounds for the research (Denscombe, 2008). Sampling was purposive with regard to sex educators’ gender, region, and length of employment, and online followers’ gender, sexual identity, and region. Data were collected between September 2022 and December 2022.

The open-ended questions were designed separately for sex educators and their followers based on the systematic literature review conducted by the research team. Interview guides covered topics such as the experience of delivering and receiving the various program components, the sex educators and young people’s engagement with these, perspectives on possible barriers to delivery, and ways to support the inclusion and delivery (for the full interview guide, see the online supplement). As the interviews followed an iterative approach, additional lines of questioning were followed based on the unique issues raised by each participant. Interviews were conducted by a single research team member to ensure consistency in question interpretation and to minimize discrepancies in interview content. The sessions were conducted online via Tencent meeting to facilitate long-distance communication from different regions of China with each session lasting from 45 to 60 min. The interview was audio-recorded with the participant’s consent and transcribed verbatim by Tencent meeting. The post-analysis translation method was utilized for retaining explicit and implicit meanings embedded in the language for culturally specific expressions and concepts (Suh et al., 2009). The research team met regularly to review the interview codes and themes until no new topics were emerging, indicating data saturation was achieved (Saunders et al., 2018). The accuracy of the transcripts was checked by the interviewer.

### Data Analysis

The qualitative data were analyzed using the software package NVivo and followed a mixed approach of thematic analysis and the constant comparative method. The thematic analysis examined the data set to find repeated patterns of meaning and helped systematize the large amounts of textual data (Terry et al., 2017). The thematic analysis process involved transcribing the data, reading and re-reading the transcripts, and conducting independent coding by two members of the research team. One coder specialized in gender studies and the other specialized in psychology, with the aim of incorporating diverse perspectives to enhance the robustness and comprehensiveness of the coding process. A codebook was established that included definitions for the relevant terms and concepts, which served as a reference guide for the coders.

The two independent coders applied the full codebook, which included all the relevant codes and definitions, to the complete data set of interview transcripts. Regular meetings were held between the coders to discuss any discrepancies, and a third coder was consulted when the need arose to reach consensus.

Following the thematic analysis, the research team then utilized the constant comparative method to further refine and develop the thematic structure. This involved the team meeting regularly to compare codes, discuss emerging themes, and reach consensus on the final set of themes that captured the key concepts in the data. The constant comparative method was used throughout the data analysis process to reexamine the codes and identify commonalities and differences (Strauss & Corbin, 1998). To ensure the researcher’s reflexivity, memos were also used to record interpretations and develop coding decisions (Levitt et al., 2018).

## Results

### Overview

The sample consisted of 28 adults (10 sex educators and 18 young followers). Of the 10 sex educators, most (N = 7) self-identified as women; half of the sex educators (N = 5) had more than three years of working experience. A total of 18 online followers (9 women, 7 men, 2 transgender) aged 18 to 26 years completed the interviews. Those young followers had a variety of sexual identities including heterosexual, gay/lesbian, bisexual, and pansexual. The demographics of the participants are presented in detail in Table 1.

**Table 1:** Sample profile of the Chinese sex educators and online followers (n = 28)

Characteristic	N
Sex educators (n = 10)	
Gender identity	
Man	3
Woman	7
Length of employment, y	
1 to < 3	5
3 to < 5	3
5 to < 10	2
Region	
Southern China	5
Northern China	5
Online followers (n = 18)	
Gender identity	
Man	7
Woman	9
Transgender	2
Sexual identity	
Heterosexual	7
Gay/lesbian	5
Bisexual	4
Pansexual	2
Regions	
Southern China	9
Northern China	9

Five overarching themes emerged: potential effect on traditional sexual roles; intra-feminist conflicts vs. cooperation around sexuality via the online realm; the role of men in a feminist sexuality education program; stigmatization of the feminist approach in China; and constitutional and political constraints on online sexuality education using a feminist approach. Study participants are identified by their identity (“E” refers to the educator and “F” refers to the follower), number, and gender (“M” and “W” refer to men and women, respectively, and “T” refers to transgender).

### Potential Impact on Questioning and Subversion of Assigned Sexual Roles

This theme came up, for example, in discussions of the current practices and impacts of social media-based sexuality education in China. A few Chinese sex educators mentioned the feminist principles in sexuality education curricula, and this approach garnered positive responses from online followers, with participants experiencing reduced shame surrounding sex and rejection of the repression of women’s sexuality and binary gender norms.

#### Affirming Women’s Entitlement to Sexual Pleasure

Some sex educators shared their experiences of integrating feminist concepts such as women’s self-determination, empowerment, and sexual pleasure in sexuality education, and indicated that this approach had received some positive feedback from their online followers. As one participant stated, “A workshop titled ‘Women on Top,’ which I facilitated a few days ago, was very much aligned with feminist principles. I encouraged women to take the lead during sexual intercourse, maintain control over the rhythm, prioritize personal pleasure, and exhibit confidence in self-expression, which were the key messages. I was able to effectively influence their behavior through these methods before changing their attitudes. Feedback from some participants revealed that after learning these techniques, they experienced a reduction in feelings of shame and stigmatization associated with sex.” (E8, M)

This view was echoed by the sex educator’s online followers. Many online followers talked about their experiences of online sexuality education, and in particular how it influenced their comprehension of sexual desire. Sexual desire had been normalized in these narratives and casual sex was seen to be regarded as a personal choice in contemporary China. As one participant described: “I think having casual sex is purely a personal choice, and it does not seem like an immoral action as many people criticized! As long as you do this kind of thing safely and with consent, it is purely personal morality and does not violate the law! As long as you are clear about what you want from the beginning, some people who want to hook up will naturally not contact you if you want to establish an intimate relationship.” (F28, M)

#### Resisting Traditional Sexual Script

The analysis revealed an emerging challenge to traditional sexual norms that had defined women’s sexuality in reactive terms. Some participants questioned the cultural suppression of women’s sexual desire, as one put it: “People may think that women should not actively express their desires. Some women do not know how to express their desires, or they feel like they do not have that strong desire or desire at all. Having sex for them is a bit like completing a task. This is all influenced by the traditional sexual norms” (F13, W). Some explicitly rejected the patriarchal norm of women’s virginity and stated that the traditional value of women’s sexual purity was employed to subordinate women, as “the vagina is the path to the soul, and if the body is not liberated, the mind cannot be opened” (E7, W). Some also suggested that chastity was no longer a symbol of honor and women were no longer the objects of men’s desire. One participant reported that she was willing to call herself “Zha nü” (bad girl) which was the opposite of “good girl” who adhered to gendered morality in sexual activities and considered it as an appraisal.

“Some people insist on having sex after marriage, believing that this is the kind of ‘good girl’ who is loyal and innocent. I often joked with my friends, saying I am a ‘zha nü,’ and they said how could you say that about yourself, and I said I did not believe that was a derogatory term.” (F16, W)

### Intra-Feminist Conflicts versus Cooperation Around Sexuality via the Online Realm

When asked about participants’ understanding of a feminist approach to sexuality education both sex educators and their online followers were unanimous in the view that there were different schools of thought, which were roughly divided into “extreme feminism” and “peaceful feminism.” Participants indicated that these two groups of feminists shared common goals, which were to subvert sexist cultures and challenge patriarchy, and both relied on social media platforms to intervene in public events. However, the ideological content of feminist practice varied.

#### “Extreme Feminism” Against Femininity and Heterosexual Marriage

“Extreme feminism” is understood as an ultimate example of feminism which promoted anti-marriage and anti-femininity agenda among a majority of participants. Participants mentioned that “extreme feminists” criticized women who used beauty products as “serving in patriarchal beauty system” and married women as “a donkey working full time for the family.” While a minority of participants had positive attitudes toward the radical performance of “extreme feminists,” all agreed that “extreme feminism” was dividing Chinese women. A number of participants accused “extreme feminism” of insulting individual women who conformed to the cultural standards of beauty ideals and desire for heterosexual marriage. One female participant stated: “I was worried that I was not qualified as a feminist and would be the target” (F18, W). And another commented: “Their collective actions which persecuted women under the cover of awakening women is not different from patriarchal practices” (F21, T). One participant also suggested that: “Women improving on their physical appearance is not necessarily for seductive purposes. For example, they might do it for a healthy lifestyle, or a sense of self-esteem. I also think feminists should respect women’s choice of intimacy or marriage, and teach them how to develop healthy relationships” (F15, W). Results showed that participants stood on the side of free will and accused “extreme feminists” of restricting women’s behaviors in ways that were the same as patriarchal disciplines which, could constitute another type of oppression and exploitation of women.

There were also some negative comments about the “essentialist” nature of “extreme feminism.” Although “extreme feminism” disseminated the idea that feminism was about equality for both genders, some participants reported that “extreme feminists” were man-haters who attacked men and argued that those feminists were only concerned with groups whose biological sex was female. However, perceptions differed as to whether biological males had more rights than biological females. Some felt that biological males of all genders indeed benefited from male privilege. For example, one participant gave an example of gay men’s oppression of women: “In general, men are active in minority groups. For example, dirty jokes dominate gay spaces and threaten lesbians. Therefore, many lesbians do not speak” (F19, W). Others acknowledged that there were gender tensions in the community, but argued that comparing the privilege or oppression experienced by certain gender and sexual identities was pointless. As one participant indicated: “Women and biological males in LGBTQ groups were all marginalized groups and were all oppressed in some ways based on their dimensions of identities. These two groups can work together on common goals of eliminating institutionalized heterosexism.” (F26, M)

#### “Real Feminism” Should Focus on the Public Sphere

Unlike the controversy surrounding “extreme feminism,” participants had a more consistent view of “peaceful feminism.” Participants drew on the concepts of equal opportunities and social justice from Western feminism to describe the core values of “peaceful feminism.” For example, one participant said: “‘Peaceful feminism’ is fighting for women’s right to employment, education, sexual and reproductive health, and equal share of domestic tasks” (F20, T). “Peaceful feminism” was recognized as truly working for women’s rights and advancing policy improvements in the public sphere rather than judging women's personal lives. However, participants also expressed a desire for more offline collective actions for women who are being treated unfairly instead of the empty slogan of uniting women on the Internet.

#### Feminist Approach to Sexuality Education Should be Inclusive and Reflexive

Commenting on how to address the internal tensions among different feminist approaches, one of the sex educators suggested: “I think feminism is a spectrum. Some people may take a more radical stance, some take a moderate stance, and some may take a compromising approach. But as long as you agree that gender equality is our goal, we can unite together. Because feminism should exhibit a very inclusive stance, it is standing on the opposite side of this patriarchal culture which contains authoritarianism and violence” (E6, W). Another sex educator also suggested reflecting on the standpoint and power relationships between sex educators and target audiences, such as examining how sex educators interpreted or defined the meaning of an individual’s sexual experiences.

“One particularly memorable instance was a young girl who desired a sexual experience and went to a movie theatre, where a man seated nearby began using intimate language and making physical advances toward her, which she willingly accepted. I was thinking that although she was an underage woman, did she possess sufficient agency to determine what she wanted and did not want? Can we judge her from our standpoint?” (E5, W)

### The Role of Men in a Feminist Sexuality Education Program

Issues related to including men in a feminist sexuality education program were prominent in the interview data. The inclusion of men was seen to pose barriers such as men showing less interest due to perceived lower responsibility and their stricter adherence to gender roles. Similarly, the inclusion of men also provided some opportunities.

#### Barriers: Gender Differences in Reporting Risk and Responsibility of Sexual Health

With respect to social media-based sexuality education, a high acceptance among women and low interest among heterosexual men was observed by both sex educators and their online followers. Some participants suggested that one of the main reasons for this might be that most of the responsibility when it comes to reproductive health, contraception, and sterilization, lies with women. As one participant commented, “As a woman, I tend to pay more attention to some special needs of women, such as the provision of sanitary napkins. However, my male friends around me have never paid any attention to this and even believe that sanitary napkins are marked with a ribbon on our underwear” (F14, W). Some participants also reported that some women actively participated in sexuality education because of their motivations to “protect” themselves from male perpetrators, as they perceived that women were at high risk of sexual violence. On the contrary, the risk and danger of sex did not resonate with men, as one sex educator reported, “There may be two core needs for men to subscribe to online sexuality education courses. The first is how to improve sexual skills, including lasting longer in bed, learning more new sex positions, and getting a harder erection. The second part is the need for social interaction. They will feel that coming into such a learning space allows them to meet some people, express their desires, or find some sexual partners.” (E8, M)

#### Barriers: The Maintenance of Hegemonic Masculinity Through a Rejection of Sexuality Education

Participants widely reported that heterosexual men were less involved in online sexuality education, with some participants assuming that it was because men were reluctant to express ignorance about sexual knowledge as they were always perceived as “knowers” in terms of sex and were unwilling to lose this authority. As one man suggested, “Perhaps influenced by traditional culture, they have a sense of confidence coming from nowhere that ‘I know all the stuff, so why do I need to learn these things?’ They want to act like they are experienced and professional” (F23, M). However, participants felt that most men lacked sexual knowledge, and pornography was the only source of sexual information for them. For example, one participant said: “I do not think they realize that their knowledge is insufficient. When I asked a friend if he knew there were any contraceptive methods, I found that the only method he knew was condom. I feel like their learning from pornography was only superficial.” (F11, W)

Participants also reported that men were more likely to be hostile toward sexual behaviors that violate traditional gender roles such as women actively exercising sexual autonomy or men having sex with men. Several participants also showed that some men dismissed the struggles of their lower-status counterparts. For example, participants reported that “regarding ‘Tangshan attack’ (a group of men attacked four women at a barbecue restaurant in the northern district of Tangshan Road), some netizens expressed a denial that sexual violence was gender-based but linked the attack to organized crime and thuggery.” (E2, W)

#### Opportunities: Men Learning Feminism Through the Process of Sexuality Education

Although there might be multiple challenges men face in engaging in feminist activities, some participants suggested the potential positive relation of certain men to feminism. Some participants in this study showed their recognition of the oppression of restrictive sexual norms placed on Chinese men. For example, some participants highlighted the pressure produced by masculine norms for perfect sexual performance and suggested focusing more on mutual pleasure rather than the frequency and timing of sexual encounters. One participant commented, “Perhaps influenced by traditional beliefs of masculinity… But in fact, you cannot always be that long about the duration. Besides, if the time is too long, girls may not necessarily feel happy. We need to consider the cooperation between both parties and adjusting for too long can also be seen as a form of torture by others” (F28, M). Participants also suggested that dominant notions of masculinity discouraged male victims of sexual assault from reporting what had happened to them and that adopting a feminist approach could help address male sexual victimization. One of the participants said, “Boys will also be subjected to sexual assault, but the situation boys face may be more severe than girls. Because boys are even more afraid to speak out and feel very ashamed influenced by patriarchal thoughts. So, I think it is better to use feminist thinking for sexuality education.” (F24, M)

Some male participants exemplified their awareness of discrimination against gender non-conforming and non-heterosexual people, by questioning on heteronormative gender roles and power imbalances. Some of them actively embraced sexual diversity well beyond tolerance, “People are inherently diverse, and it is impossible for one gender to be tied to a label, right? A label is just an adjective, and you cannot say that using this standard to demand everyone, no individual of any physiological gender can become a true winner” (F23, M). Some further criticized heteronormative patterns in which women were placed in a submissive position and men generally played an active courter role. For example, one participant commented, “I disagree with the idea of men’s aggressive sexual behaviors due to purely physical reasons such as hormones. I feel like they are using unchangeable factors to explain this. I think culture can exaggerate gender differences, and most importantly, the power, for example, men as a dominant group having the structural power.” (E10, M)

Some male participants expressed a positive view on women’s sexual desire. One participant suggested that women had the same right to pursue sexual pleasure as men and commented that: “Boys’ masturbation is also something they can solve themselves. I think both boys and girls should have their unique ways to explore pleasure” (F28, M). Another participant alluded to the notion of concession when he saw her girlfriend enjoying self-sexual exploration and commented: “At first, I felt a bit sad and hurt. But after communicating with my girlfriend, I did not think it was a big deal. Because the psychological intimacy such as the pleasure of we are holding together, cannot be replaced.” (F23, M) When asked about how to subvert the stereotypical sex roles of active men and reactive women, one participant suggested that “femdom” might work as gender reconstruction practices but added that classical heterosexual partnership with feminine dominants and masculine sub-missives was still not ideal.

#### Young People’s Suggestions to Maximize Men’s Involvement

Some young people suggested emphasizing gender and sexual diversity when conducting a sexuality education program, for example, highlighting the importance of recruiting across all genders, sexual orientations, and nationalities. Some participants also suggested that the issues of “intersectionality” in which everyone as a minority was affected by interlocking systems of power should be addressed during sexuality education. Others also considered establishing a platform for men’s self-defense and vulnerability which could address the concerns raised where sexuality education was seen as oriented toward women’s interests and concerns. For example, it could be a platform for biological men to express their feelings of being attacked as a member of men’s groups when it comes to gender-based violence or sexually abused men to talk about their abuse. While a minority insisted men and women constituted opposing sides, all agreed that men can be educated to change their beliefs of gender and sexuality and go on to understand women’s dilemmas, needs, and concerns. As one participant put it, “I have noticed that some of my male friends are doing self-learning in sexuality. I have observed that they are all proactive in obtaining updated information. Then I have a feeling that I am educating them, and I feel that they are indeed making progress.” (F13, W)

### Stigmatization of the Feminist Approach to Sexuality Education in China

Both sex educators and their online followers were uneasy about the label of “feminism” when asked about their views on using a feminist approach to sexuality education. They agreed with the common goals of the feminist approach, but suggested naming sexuality education programs as based on an approach focused on core values and beliefs, rather than explicitly labeling it as a “feminist” approach.

Social prejudice against the term “feminism” could be the main factor influencing the participants’ decision to avoid identifying themselves as feminists in China, despite all participants reaching an agreement on the legitimacy of securing women’s rights. Some explained that the term “feminism” was misperceived as opposing the rights of men and establishing a woman-dominated world, which could be a mirror duplication of patriarchy. As one participant commented:

“Feminism itself, in our view, pursues the rights of a gender group represented by women in a patriarchal society, rather than replacing men. However, what a few of them advocate is to overthrow men. Just like a friend of mine who talked about his father being a very strict and patriarchal parent, and she told me, ‘I have grown up now, I have made money, and I just don't provide that old man with living expenses. I will treat him the same way he treated me back then.’” (F23, M)

Some also reported that “feminism” could be misinterpreted by some online users as a group seeking special favors when women and men already had equal rights. Participants drew on an online popular discourse “nü quan” to describe how online users understood and stigmatized women demanding rights. “nü quan” means “boxing” and shares a similar pronunciation with “feminism” in Chinese. Those groups used this pun to make fun of women who were aggressively speaking for themselves, by equating them with insane militants. One participant was also appalled by some men “labeling all actions for women's rights as provoking gender contradiction.” (F20, T)

Some participants in this study refused the label of “feminism” due to concerns about not being objective enough. “Feminism” was considered biased and participants preferred to hear scientific facts from a biomedical perspective. Participants kept emphasizing the importance of objectivity and implicitly expressed the notion that feminism emphasized the superiority of women rather than gender neutrality. As one participant said, “We talk about equal rights rather than feminism, because I am a person who does not particularly lean toward one side. When I look at a problem, I do not necessarily say this way, I may be more neutral. If you ask me to fully support something, it may make me feel unsafe. But I will agree with a large part of the views of feminism.” (E2, W)

Of note is that some Chinese sex educators strategically chose alternative terms such as “egalitarian” or “women’s rights” to describe their approach, despite their agreement with feminist values, due to public anti-feminist rhetoric in China. The comments below illustrated the safety concerns of fully supporting feminism faced by sex educators as well as their strategies for surviving stigma: "The current discussion on gender will still be quite severe. If we are labeled as extremists and reported, things will become very troublesome. Therefore, we do not have a clear identification, but we support feminism because it is a struggle for women’s rights, for gender equality, and we will also avoid touching some controversial feminist issues that will affect the unity of our community.” (E1, W)

Participants expressed fear of being “labeled” and “reported,” which refers to the act of being reported to authorities as engaging in activities deemed undesirable or subversive. There can be serious consequences when participants are reported, often by other individuals or even automated monitoring systems.

However, some participants disagreed with introducing alternative terms to “feminism.” Some other participants further indicated that it was necessary to mention “feminism” in a society where men and women were still unequal and did not see fighting for women’s rights as a radical performance. Some also suggested that a precise choice of wording was needed to ensure consistency in such processes as research documentation. One commented: “For some scholars, they may need more precise language to summarize the specific things they want to express, such as research methods or theories.” (E3, W)

### Constitutional and Political Constraints on Online Sexuality Education Using a Feminist Approach

A recurrent theme in the interviews was a sense among participants of the state’s increased censorship around issues of gender and sexuality. Some participants reported that educational content related to gender and sexuality was heavily censored using information filtering that forbade sensitive words or topics such as “gay” or “lesbian,” large-scale bans on feminist or sexual minority related organizations, tracing movements and internet records of people involved in feminist activities.

“In the past few years, I have noticed that the policy resistance was growing. The official account of sexual minorities in the past few years has been very active. For example, many universities have established relevant groups or official accounts. But in 2019 or 2020, these official accounts were banned collectively. All offline-related activities were also stopped. If they want to conduct any activities, words such as gay and lesbian, and ‘rainbow flag’ are not allowed.” (E3, W)

When asked about the reason why feminist discourses of gender and sexuality were subject to increased state means of control, the majority of participants, especially sex educators, were reluctant to discuss this at first. A few, mostly feminist or LGBTQ activists, expressed the sensitivity of this topic and finally spoke freely after having repeatedly confirmed the anonymity and confidentiality of the interviews.

#### Political Control over Discursive Rights of the Netizen

Participants suggested that the authorities attempted to maintain hegemonic discourse in online spaces to manipulate the minds of young people and blocked or marginalized other dissonant voices through central control. However, unlike traditional media that could determine which voice to be heard, the control over content in cyberspace might not work. As one person put it, “When I was young, my grandparents believed more in the information publicized by the mainstream media, such as AIDS and various sexually transmitted diseases. They seldom suspected the official view of sex as they had fewer choices of sources. But now the mainstream media is no longer the dominant source of information and there cannot only be one type of perception” (E3, W). Participants also indicated that the state was concerned about public discontent and its potential to bring about political change. A participant mentioned that the state had blocked discussions on the persecution of women and sexual minorities to avoid escalating gender issues into class conflicts and bringing about the overthrow of political power, “Some activists posting articles about people being persecuted due to their gender may attract the attention of some groups, which may lead to public dissatisfaction and the government may feel that this is not conducive to social stability” (F25, M). On the positive side, participants indicated that the public discussion on feminist, gender, and sexuality issues was not completely banned by the state which in turn had the potential to provide new opportunities for sex educators to apply the feminist approach to mobilize sexuality education.

#### Feminism as the Violation of Established Gender Order

A few participants expressed that feminists’ advocacy of women’s reproductive freedom conflicted with national policies on marriage and childbirth which could also be a barrier to implementing sexuality education using a feminist approach. Participants reported that the official account of the state labeled unmarried and infertile individuals as “extreme feminism” and a threat to political stability. However, most participants expressed disagreement with the official statement and criticized the traditional beliefs that the value of women lies in their fertility and the propagation of the future instead of emphasizing the basic rights of Chinese women over their bodily autonomy. Some participants also indicated that the reduced adherence to traditional gender roles among young people could also be a concern for authorities. Participants mentioned that the Education Ministry of China had advised schools to “cultivate masculinity” and promoted “avoiding feminization” for its schoolboys. Participants also felt that the state was gradually encouraging traditional masculinity and femininity and that consolidating the patriarchal socio-economic structure that favored male dominance.

#### Feminist Sex Educators Can Take Advantage of Digital Platforms

Digital feminist sex educators have been trying to respond in creative and innovative ways to evade control and share their perspectives. Some participants reported that sex educators would circumvent censorship by replacing sensitive words with symbols or homonyms, not publicly sharing information about the time and place of activities, pixelating some sensitive pictures, using backup accounts, and deleting content regularly. The tactics employed by feminists to counter online censorship also included disguising their activities within the state-mandated political agenda for health promotion within equality education, family harmony, education for women’s self-confidence, and protection or STI prevention initiatives.

“For example, when we see the newly revised Women’s Rights Protection Law, we think that including equality education will become a political endorsement for our activities. Then, we can only use this banner to carry out activities, or we will use the simplest way to emphasize gender equality and minimize the use of ‘feminism’.” (F23, M)

“The lesbian group has now organized many activities with the topic of ‘protecting women’, which is how the activities can continue. It cannot be said that there are sexual minorities, but rather a gimmick to protect women and improve their confidence.” (F19, W)

Some sex educators also reported that the anonymity of the Internet had helped create a gender-friendly teaching environment. They indicated that young people actively shared and discussed diverse sexual experiences on social media platforms such as WeChat and Weibo. Furthermore, they noted that sex educators could teach content beyond physiology and health science due to less social resistance and scrutiny. For example, some educators reported that they were able to teach intimate relationships online, including the interplay between love and sexual techniques to enhance mutual sexual satisfaction. Additionally, some educators reported that pornography could be covered, with a focus on dispelling misinformation surrounding unprotected sex and challenging unrealistic body expectations. The sexual experiences of women could also be addressed as suggested by some participants, with discussions centered around crucial topics such as abortion rights, combatting menstrual shame, and managing emotional dysregulation caused by hormonal fluctuations during the menstrual cycle. Furthermore, participants highlighted the potential for introducing sexual identity and orientation by leveraging upon relevant social events for transgender individuals and engaging in discussions about the legislation of same-sex marriage. However, some young followers also expressed concerns about the lack of assessment of teaching quality. Some of them also reported that they were unwilling to post negative comments even if they disagreed with the content. These results suggest that the intersectional power relationships between sex educators, target audiences, and government scrutiny still need to be considered.

## Discussion

This study is among the first to explore the perceptions of both sex educators and their online followers regarding social media-based sexuality education using a feminist approach in China. Prior research has highlighted the limitations of the current mainstream traditional, biomedical-focused sexuality education programs, which often fail to address the broader social and relational aspects of sexuality (Cacciatore et al., 2019; Ketting et al., 2021; Leung et al., 2019). In contrast, a feminist approach to sexuality education may challenge power imbalances and promote a more holistic, empowering understanding of sexuality (Connell & Elliott, 2009; Harrison & Ollis, 2015). The current study suggests that social media may serve as a strategic platform for implementing this approach in China, where traditional sex education approaches remain inadequate and limited.

Results suggested that (1) Chinese young people in this study expressed positive attitudes toward integrating feminist concepts in sexuality education and with perceived benefits including helping young people, especially young women, to overcome a sense of shame, challenging sexual double standards, and improving their expressions of sexual desire; (2) feminist approaches to sexuality education in China could be seen to have evolved from dual influences of Western feminism and inter-Asian feminist movements. This has generally developed in two broad directions, “extreme feminism” and “peaceful feminism.” Although the two differed in ideological content, an emphasis on the spectrum of various stances had emerged. Paying attention to inclusive feminist approaches and power relationships between sex educators and their audience may also help address the resulting divisions; (3) little relevance of educational content and acknowledged male privilege characterized the barriers to Chinese young men’s involvement in the program. Despite the challenges men face in engaging with feminist activities, our study revealed that the men interviewed recognized the oppression resulting from restrictive sexual norms on men and exhibited a potentially positive relationship toward feminism; (4) sex educators and their online followers in China were observed to be hesitant to adopt the label of “feminism” in relation to their sexuality education programs, due to negative social prejudices against the term. They recognized the legitimacy of securing women’s rights, and the stigma associated with feminism had led to the choice of strategically more moderate terms. Of note some participants argued that feminism must be explicitly mentioned to fight for gender equality and the need for social recognition; and (5) despite state censorship and attempts to maintain hegemonic discourse around gender and sexuality issues, digital feminist sex educators used creative and innovative tactics to evade control and promote their ideas, including replacing sensitive wording with symbols and homonyms, not publicly sharing information about activities, and disguising their message under the guise of health and family harmony initiatives to align with Chinese government policies.

The current study showed that adopting a feminist approach to sexual education might serve to affirm women’s sexual pleasure and had the potential to subvert gendered presumptions of women’s passivity and victimhood, which enable behavioral change. This aligns with previous research, which highlighted the transformative power of feminist frameworks in sexuality education (Askew, 2007, Bay-Cheng & Lewis, 2006; Kolenz & Branfman, 2019). Moreover, our findings uncovered additional insights that extend beyond prior studies, emphasizing that directly teaching young people about sexual behaviors, such as the “women on the top” posture, could enhance women’s sensory arousal and familiarity with their bodies. This approach might in turn help young people change their knowledge and attitude toward sexual pleasure. This result should be taken into account when considering the inclusion of the content of sensual pleasure and desire in the sexuality education curriculum.

Our investigation into the broader feminist landscape in China reveals significant divisions and diverse intentions among feminists themselves, which aligns with findings from previous study (Hong et al., 2022; Yang & Zhou, 2023). The “extreme feminism” in China appears to have been influenced by both Western radical feminism as well as inter-Asian influences, such as the 4B movement in South Korea, which encompass principles opposing traditional heterosexual marriages, femininity norms, and procreation (Lee & Jeong, 2021). Its anti-marriage and anti-femininity claims were considered to provoke antagonism and split up existing women groups. This finding is consistent with prior research indicating that some feminists in China disproportionately targeted women themselves, creating an environment that burdened and stigmatized women for their choices (Chang et al., 2016; Wu & Dong, 2019; Yang & Zhou, 2023). Our unanticipated finding was that some Chinese sex educators were not blindly denying “extreme feminism,” but rather affirming its influence in making some deeply rooted gender issues visible and highlighting the necessity of their existence within the spectrum of feminism. While the previous studies have focused on the criticism of “extreme feminism” (Mo, 2022; Zhao et al., 2022), the current study contributed to a clearer understanding of Chinese young people’s complex attitudes toward Chinese feminism and their prioritization of unity, diversity, and mobilization of women and sexual minorities.

On the other hand, “peaceful feminism” might align with “liberal feminism” portrayed in Western societies. Our results were broadly in line with previous studies (Ma, 2022; Shao, 2023; Yang & Zhou, 2023) that Chinese young people were more likely to espouse a liberal perspective of feminism which embraced the Western agenda of autonomy, freedom of choice, as well as equal rights and opportunities as the standard. Moreover, this study revealed that young adults in China expressed a desire for “peaceful feminists” to employ strategic approaches aimed at advancing policy improvements and mobilizing collective actions within the public sphere. However, participants noted a lack of tangible progress in these areas. This observation aligns with recent literature, which suggested that the current political climate in China poses significant challenges to the integration of feminist concepts into public discourse (Zeng, 2019).

The ongoing gender anxiety and proliferating discussion on feminism in contemporary China have raised concerns about the relationship of men to feminist discourses of sexuality. The data suggested that men’s attitudes toward sexuality education ranged from disinterest, rejection, to support, and these attitudes were aligned with their positioning toward feminism. A similar pattern of results was obtained in Crowe’s (2011) study which classified men’s response to feminism as “not about them,” opposing their interests and exhibiting their active support.

Regarding the negative attitudes toward feminism, our findings were in accord with previous studies indicating that gender equality historically as a topic of concern primarily to women’s and the privileges of hegemonic masculinity could influence men’s intentions to support gender equality (Crowe, 2011; Holter, 2014; Limmer, 2010; Lingard & Douglas, 1999; Newton, 2002; Pleasants, 2011). For example, male participants’ emphasis on improving sexual skills, lasting longer in bed, learning new sex positions, and getting a harder erection aligns with the idea of hegemonic masculinity. Men may feel the need to subscribe to online sexuality education courses in order to fulfill these expectations and enhance their sexual abilities, which are often associated with asserting a sense of manhood and power (Groes-Green, 2009). This poses challenges to conducting feminist sexuality education and contradicts the principles of equality and mutual pleasure.

Nonetheless, quite a number of male participants further questioned the pervasiveness of the current gendered power structures in China rather than simply questioning stereotypical gender roles and discriminatory practices. The findings offer a more hopeful perspective, suggesting that some men, traditionally seen as oppressors, can indeed comprehend the actual circumstances of the oppressed and grasp the nature of structural oppression (Zhao et al., 2022). A possible explanation for this might be that the volunteers for interviews had a higher degree of prior interest in feminism. Participants also offered some advice to involve men in the sexuality education program, such as establishing a platform for men’s self-defense and expression of vulnerability and emphasizing diverse gender identities, when promoting the program. These results provide a new insight when considering men’s role in fostering gender equality and fighting against sexism or oppression.

The stigmatization of feminism is also a pervasive theme across the analysis. In line with previous studies conducted in China, the Chinese feminist approach could be misinterpreted as an ideology for promoting women’s privileges which hindered feminists’ struggles for emancipation and degraded women to return them to an inferior social position (Huang, 2023; Sudo & Hill, 2006). The possible reason behind was perceived as the overly subjective nature of feminist thoughts. Participants might have been influenced by a masculine notion of objectivity which assessed feminism through an assumed position of non-bias and discounted the validity of women’s experiences (Nelson, 1996; Pleasants, 2011). In response to the stereotypical image of feminist concepts, we put forward the strategies Chinese online sex educators used to cope with and manage these situations, such as framing their sexuality education as “egalitarian” to maintain socially accepted ways of discussing gender inequality, celebrating feminist identities, or using “feminism” only as a technical term. These findings have extended our knowledge of feminist tactics for cultivating safe spaces for sexuality education in China.

The interviews also revealed significant external barriers such as political pressures to effectively implementing sexuality education using a feminist approach in China. The state’s concerns about legitimacy and maintaining social stability contributed to the increased control over feminist approaches to gender and sexuality (Wu & Dong, 2019). This is particularly relevant in the context of President Xi’s emphasis on upholding Chinese traditional family virtues as part of the party’s “women-related work” (Wallis, 2015; Yin, 2022; Yin & Sun, 2021; Zeng, 2019). Most participants believed that the rigorous restrictions on freedom of expression and pervasive monitoring of the internet severely constrained citizens’ capacity to engage in any form of opposition against China's authoritarian leadership which matches those observed in a previous study (Liao, 2020). The repercussions included the deletion of their online posts, suspension or termination of their accounts on social media platforms, and even permanent banishment from these platforms (Han, 2018; Roberts, 2018; Wang & Driscoll, 2019; Xu et al., 2011). Moreover, feminists may encounter personal attacks from nationalists who perceive feminism as morally destructive and as an imported concept linked to a “bourgeois” project associated with Western influence (Huang, 2018). These attacks can lead to the removal of their online presence and severed contact with their supporters, further isolating them within the digital space (Dian, 2023).

Although feminist activists faced remarkable obstacles in creating discursive rights and challenging existing political power and social order within a tightly controlled space (Sun & Yin, 2022), they found ways to leverage social media platforms like Weibo and WeChat to raise awareness about gender issues in China (Chen et al., 2020; Wang & Driscoll, 2019; Yang & Zhou, 2023). The relationship between censors and netizens can be described as a continuous cat-and-mouse game, where netizens employ creative strategies to evade censorship (Endeshaw, 2004; Esarey & Xiao, 2008). Activists utilize tactics such as avoiding controversial posts, practicing self-censorship, employing memes, hashtags, and emojis, and carefully managing their social media accounts (Dian, 2023). Interestingly, Chinese sex educators also drew inspiration from the discourses of authority to carve out legitimate space within the realm of sexual politics, as described by Chen (2019) as “utilizing the government’s agenda for their own goals.” For example, they disguised themselves under the political agenda of health promotion in the name of equality education, family harmony, women’s self-confidence, or HIV prevention to circumvent censorship. This finding offered a new strategy for bypassing local censorship when spreading feminist values in online sexuality education programs.

Our findings also highlighted the positive aspects of anonymity of the Internet in facilitating open communication and expanding teaching content. This aligns with media studies that the anonymity provided by the Internet can lead to more open self-expression and reduced social resistance, creating a safe space for discussing sensitive topics and challenging societal norms (Atkinson & DePalma, 2008; Qiang, 2011; Van Heijningen & Van Clief, 2017). Our findings also shed light on the potential challenges related to power dynamics and the need for assessment and feedback mechanisms in online educational spaces. This is consistent with the previous studies of the digital divide and power differentials in online spaces which emphasize the importance of considering power relationships in online interactions (Agarwal et al., 2009; Riggins & Dewan, 2005; Van Deursen & Van Dijk, 2014).

### Implications

Firstly, our study sheds light on the lack of attention given to the ideological content of feminist practices and the limited public understanding of feminism in China. This knowledge gap creates a disconnect between individuals advocating for gender equality and feminists, despite sharing similar goals. Furthermore, our research acknowledges the presence of hostile attitudes toward feminism in China, from both the state and the public, which contributes to an unfavorable or even toxic environment for feminists.

At the same time, our study also recognizes the resilience and power of feminist resistance in China. Feminists in our study have found ways to navigate the constraints, utilizing social media platforms to raise awareness and engage with gender issues. The anonymity provided by online spaces also facilitates open discussions around sensitive topics, creating a safer environment for challenging societal gender norms. In addition, we observe that the younger generation’s concerns about gender and sexuality, as well as their rejection and critique of traditional norms, present possibilities for the adoption and transformative impact of feminist approaches in China. More research is needed to understand the specific strategies and support structures that can help sex educators navigate the constraints they face.

### Limitations

Our research sample aimed to feature diversity in terms of gender, sexual identities, and geographic regions in order to avoid potential biases inherent to demographically homogeneous samples. Despite this effort, the sex educators in the current study were mostly women, likely due to self-selection bias. Additionally, most participants were generally supportive of incorporating feminist values into social media-based sexuality education. Therefore, our findings may be more applicable for populations that already have some familiarity with feminist perspectives.

While these factors represent limitations of the study, the results still provide important evidence supporting the potential benefits of a feminist approach to sexuality education, particularly when leveraging the unique affordances of social media platforms.

### Conclusion

This study is the first to explore the Chinese young people’s attitudes toward incorporating a feminist approach into social media-based sexuality education. The themes suggest that adopting feminist perspectives into social media-based sexuality education may have the potential to reduce sexual shame and increase positive attitudes toward women’s sexuality. At the same time, this approach also needs to address issues such as internal conflicts within the feminist discourse, unbalanced gender distribution in attendance in sexuality education courses, social stigmatization toward feminism, and political constraints on promoting gender and sexuality issues in China. Our findings shed light on how current sexuality education practices can take advantage of digital media to promote gender equality initiatives.

## Supplementary Information

Below is the link to the electronic supplementary material.

Supplementary file1 (DOCX 16 kb)

## Data Availability

The participants of this study did not give written consent for their data to be shared publicly, so due to the sensitive nature of the research supporting data are not available.
