# Supplementary material for: Perspectives of Young People on Social Media-Based Sexuality Education Using a Feminist Approach in China: A Qualitative Study
Source: Arch Sex Behav. 2024 Nov 18;54(2):589–604. doi: 10.1007/s10508-024-03015-z (PMC11836079; doi:10.1007/s10508-024-03015-z)
Supplement: Supplementary file 1 — Supplementary file1 (DOCX 16 kb) [file 10508_2024_3015_MOESM1_ESM.docx]

Supplementary material:

**Semi-structured interview guide (for sex educators)**

The following open-ended questions will be posed to the study participants and based

on their responses further probing questions will be asked.

**Participants’ information**

| Participant No. |
| --- |
| Gender |
| Length of employment |
| Geographical location |

**Part one: Experiences of teaching sexuality education through social media**

1. Tell me about your sexuality education program.

2. Why do you choose social media as a platform to deliver sexuality education?

3. How do you teach sexuality education through social media? What pedagogical

approaches/strategies do you use to make young people engage in the lessons?

4. How do you derive the contents in your sexuality education program?

5. How do you evaluate your sexuality education lessons?

6. What difficulties do you experience in teaching sexuality education on social

media? How do you deal with those difficulties?

**Part Two: Views of incorporating feminist perspectives into sexuality education**

7. What is your view on feminism?

8. What do you think of teaching sexuality education using a feminist approach in

China?

9. What are the advantages and disadvantages of teaching sexuality education using a

feminist approach in China?

10. What are the opportunities and challenges of teaching sexuality education using a

feminist approach in China?

11. What are your views on social media as a platform for sexuality education using a

feminist approach in China compared to the traditional methods?

12. Do you have any suggestions on implementing sexuality education using a feminist

approach on social media in China?

**Semi-structured interview guide (for online followers)**

The following open-ended questions will be posed to the study participants and based

on their responses further probing questions will be asked.

**Participants’ information**

| Participant No. |
| --- |
| Gender |
| Sexual identity |
| Geographical location |

**Part one: Experiences of attending social media-based sexuality education**

1. Tell me about your experiences of receiving sexuality education.

2. How do you find sexuality information?

3. How do you decide which sexuality information is accurate and applicable to

you?

4. Do you engage in the social media-based sexuality education program?

5. What do you think of the role of educators/facilitators/mentors in such sex

education programs?

6. How do you know you have learned something useful to you?

7. How do you think the programs could be improved?

**Part two: Acceptability of feminist messages/values/ideologies in sexuality education**

8. What is your view on feminism?

9. What do you think of feminist thoughts/values in sexuality education?

10. What are you most interested in sexuality education using a feminist approach?

11. How do feel about the way sexuality education using a feminist approach should be delivered?

12. Do you have any suggestions on implementing sexuality education using a feminist

approach on social media in China?
